# A Standardized Dataset of a Spontaneous Adverse Event Reporting System

**DOI:** 10.3390/healthcare10030420

**Published:** 2022-02-23

**Authors:** Mohammad Ali Khaleel, Amer Hayat Khan, Siti Maisharah Sheikh Ghadzi, Azreen Syazril Adnan, Qasem M. Abdallah

**Affiliations:** 1 Discipline of Clinical Pharmacy, School of Pharmaceutical Sciences, Universiti Sains Malaysia, Gelugor 11800, Penang, Malaysia; maisharah@usm.my; 2 Advanced Medical & Dental Institute, Universiti Sains Malaysia, Bertam, Kepala Batas 13200, Penang, Malaysia; drazreenadnan@gmail.com; 3 Department of Pharmacology and Biomedical Sciences, Faculty of Pharmacy and Medical Sciences, University of Petra, Amman 11196, Jordan; qasem.abdallah@uop.edu.jo

**Keywords:** FAERS, LAERS, adverse drug reactions, spontaneous adverse event reporting, ROR, PRR, information component, drug adverse event

## Abstract

One of the largest spontaneous adverse events reporting databases in the world is the Food and Drug Administration (FDA) Adverse Event Reporting System (FAERS). Unfortunately, researchers face many obstacles in analyzing data from the FAERS database. One of the major obstacles is the unstructured entry of drug names into the FAERS, as reporters might use generic names or trade names with different naming structures from all over the world and, in some cases, with typographical errors. Moreover, report duplication is a known problem in spontaneous adverse event-reporting systems, including the FAERS database. Hence, thorough text processing for database entries, especially drug name entries, coupled with a practical case-deduplication logic, is a prerequisite to analyze the database, which is a time- and resource-consuming procedure. In this study, we provide a clean, deduplicated, and ready-to-import dataset into any relational database management software of the FAERS database up to September 2021. Drug names are standardized to the RxNorm vocabulary and normalized to the single active ingredient level. Moreover, a pre-calculated disproportionate analysis is provided, which includes the reporting odds ratio (ROR), proportional reporting ratio (PRR), Chi-squared analysis with Yates correction ($x^{2}$), and information component (IC) for each drug-adverse event pair in the database.

## 1. Introduction

Drug post-marketing surveillance programs aim to minimize the risk of drug harm in clinical and pharmacy practices. It basically relies on spontaneous adverse event reporting systems. The information obtained during the drug development phase is commonly incomplete because clinical trials are limited by the short duration of drug exposure and the limited size and diversity of the population being tested. Hence, some rare adverse drug reactions cannot be linked to the drugs being tested in clinical trials, as only a few thousand volunteers are being recruited and only the more common side effects will be detected. An essential tool of post-marketing monitoring is the spontaneous adverse event reporting systems [1,2,3,4], which is an inexpensive and widely used tool in detecting new, rare, and severe adverse drug reactions [5]. Moreover, spontaneous adverse event reports can effectively detect serious adverse drug reactions resulting from drug–drug interactions [6].

It is common practice in each country to have a designated official entity responsible for supervising drug post-marketing surveillance activities [4]. In the United States of America, the Food and Drug Administration (FDA) controls the drug post-marketing safety surveillance through the FDA Adverse Event Reporting System (FAERS) [2,7,8].

Unfortunately, there are many known obstacles in using the FAERS/ Legacy AERS (LAERS) database, the most important being the unstructured entry of drug names and the uncontrolled case duplication in the database [9,10]. Drug names reported into FAERS/LAERS may have a variety of alternative naming structures, active ingredients, strengths, units of strength, dosage forms, routes of administration, manufacturing company names, and even typographical errors in some cases. Hence, the same drug entity in the database can be found in different formats, making it impossible to aggregate all these different drug name formats into a single drug entity without proper data wrangling and cleaning ahead, followed by drug names mapping and standardization to a proper drug nomenclature vocabulary. Additionally, because the FAERS/LAERS database receives reports from worldwide sources, retrieving the active ingredient names from different multinational trade names is another challenge for researchers.

Case report duplication is a known issue in spontaneous adverse event reporting databases [11], including the FAERS/LAERS database, due to the uncontrolled inputs of reports from different sources (i.e., healthcare professionals, patients, or manufacturing companies), which means the same case report can be reported multiple times from different sources. In addition, a single case report could have multiple entries representing follow-up reports of the same original case, contributing to the duplication of the case reports.

If not properly handled and mitigated to the minimum, all these issues would affect the quality and reliability of the data analysis to be performed on the database. So, a multi-step and time-consuming raw data processing should be performed first, which would require skilled people to query the database to finally obtain a clean, normalized, and standardized dataset ready to be used by any researcher. However, previous publications have addressed these issues. Poluzzi et al. introduced a method for mapping drug names and handling missing entries and case duplication for the FAERS database [10]. Banda et al. introduced a clean dataset for the FAERS database [12]. The work by Banda et al. included multi-ingredient drugs as one entity in the dataset, which may be considered as a limitation in the dataset. To the best of the authors’ knowledge, the data set of Banda et al. is the latest publicly available clean dataset of FAERS database up to June 2015. Since then, many new drugs have emerged in the market as well as millions of new case reports have been added to the FAERS database.

## 2. Materials and Methods

### 2.1. Design

FAERS is a database for the spontaneous reporting of adverse events and medication errors involving human drugs and therapeutic biological products [13]. Currently, the FDA receives over 1 million adverse-event and medication error reports of drug or biological product use annually, making it one of the largest pharmacovigilant databases in the world. Many researchers [8,14,15,16,17,18,19] prefer to use the FAERS database for pharmacovigilance data mining and signal detection, not only because of its huge number of reports, but also because it is publicly accessible, contains reports dating back to 2004, in the English language, and receives reports from all over the world, as 28% of reports are coming from countries other than the United States of America. The sources of these reports are mainly patients themselves, health providers, pharmaceutical suppliers, and manufacturers. FAERS commenced on 10 September 2012, succeeding the Adverse Event Reporting System (Legacy AERS or LAERS) publicly available since 2004. To provide a clean dataset of FAERS, two critical steps must be addressed properly: case report deduplication and standardizing drug names.

### 2.2. Downloading Source Files

The quarterly data extracts for FAERS/LAERS were downloaded for the period from the first quarter of 2004 (2004Q1) to the third quarter of 2021 (2021Q3), from the FDA’s website: https://fis.fda.gov/extensions/FPD-QDE-FAERS/FPD-QDE-FAERS.html (Accessed on 12 December 2021)

ASCII files were used as the data sources for the FAERS/LAERS database. All data files were loaded into SQL server 2019, where all data manipulations were performed, except for the manual drug mapping, for which Microsoft Access 2019 was used.

**Figure 1:**
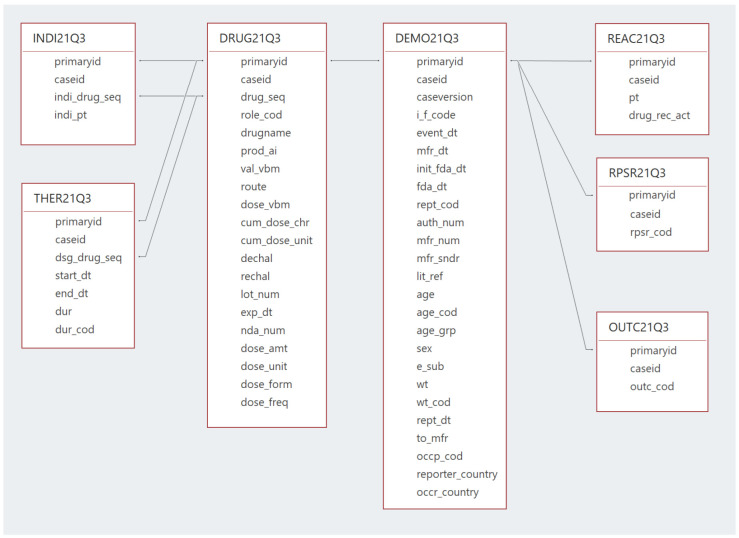
FAERS database structure and relationships.

Each quarterly data extract contains seven files, comprising the database, as illustrated in Figure 1.

The DEMO file contains the basic demographic information for the patients, where each record in this table represents a single report in the database. The DRUG file contains drug information, with the most important field being the “DrugName” as it contains the trade names or the active constituents of the reported drugs. As of the third quarter of 2014 (14Q3), a separate active ingredient or “prod_ai” field was added to the DRUG file, in addition to the “DrugName” field.

The REAC file provides the adverse drug reactions, where it includes the “P.T.” field or the “Preferred Term” level terminology from the Medical Dictionary for Regulatory Activities (MedDRA) [20], as it is used as the medical descriptor of the adverse drug events used in FAERS/LAERS. The INDI file contains the drug indications, which also uses the “P.T.” level of MedDRA as a descriptor for the drug indication. The OUTC file provides information on the outcomes of the cases, the THER file gives the start and end dates of therapy, and finally, the RPSR file contains the sources of the reported events. For each report in the DEMO file, at least one report must be available in the DRUG file and one report in the REAC file, while other files do not always have information on each case report in the DEMO file. All these files are linked together to form one database through a common primary key, which is the “ISR” for the period of January 2004 to September 2012 (i.e., LAERS) and the “primaryid” since October 2021 (i.e., FAERS). 

Since only a few differences exist between the FAERS and LAERS datasets, all the data of the same file types of the two datasets were merged into one combined table, making a unified dataset.

The ISR and “primaryid” fields were consolidated into one common field, “primaryid”, as both fields are the primary link field for data files in LAERS and FAERS, respectively.

The “PERIOD” field was also introduced, which allows the user to track any report back to its original year and quarter.

### 2.3. Case Reports Deduplication

As recommended by the FDA, a deduplication step was performed to retain the most recent report for each case with the same case identifier.

Moreover, a second deduplication process is performed, focusing on the case reports duplicated as a result of reporting the same case report from multiple sources. These reports have different case identifiers; hence, a deduplication logic was applied based on comparing and matching specific fields of all case reports.

Poluzzi et al. [10] and Banda et al. [12] assumed that if two or more case reports had the same reporting country, gender, event date, age, adverse events, and drugs prescribed, then they are most likely the same case and needed to be deduplicated.

Of all the FAERS/LAERS database cases, about 11% of the gender entries are null or not specified, and 54% are females. So, around 54% of the cases already matched in terms of the gender criteria, compromising the gender criterion’s ability to detect duplicate entries. The same concept also applies to the reporting country criterion, in which about 7% of the cases are missing the reporting country entry, and about 65% of the cases are from the United States of America, making 65% of the cases already matched in terms of the reporting country criterion. Table 1 shows the top 20 reporting countries in the FAERS/LAERS database up to the third quarter of 2021.

Missing data also impair the ability of the field “event_dt” (event date) as a deduplication criterion, as about 46% of reports have missing “event_dt”. The age is also affected by the missing entries, as about 40% of the cases are missing the age entries.

To improve the quality of the matching criteria and case deduplication, two more criteria were added to the previous ones, the therapy “start_dt” (start date) and the drug indication fields. 

However, the therapy “start_dt” was considered to be missing if no “start_dt” is found in the “THER_ combined” file as per distinct “primaryid” selection. This was found to be about 42%. The overlapping missing dates of either the therapy “start_dt” or the “event_dt” is approximately 32% across all case reports, making at least one date is present in the matching criteria in approximately 68% of cases.

Due to the enormous diversity of the administered drugs and the resulting adverse events, and the practical absence of missing data in these two fields (except for a few data entry errors), these two criteria can be considered the most important matching criteria in this deduplication step.

Moreover, the inclusion of the drug indication field in the deduplication logic would also enhance the deduplication efficiency due to the diversity of drug indication and also due to the low missing percentage in this field, as it is about 12%.

Differently than Poluzzi et al. and Banda et al., the authors considered two cases to be the same if they had a complete match of the eight criteria which are gender, age, reporting country, event date, start date, drug indications, drugs administered, and adverse reactions. Two records were also considered duplicated if they mismatch in only one of the gender, age, reporting country, event date, start date, or drug indications fields, but not the drug or adverse event fields.

The authors acknowledge that the deduplication logic, like all other deduplication logics, might have some possible mistakes, but hopefully kept it at a minimum.

Finally, starting from the first quarter of 2019, each quarterly data extract included a deleted case file, which was also used to delete the available cases in the dataset.

The count of the unique cases after the deduplication process was 11,631,635 case reports.

**Figure 2:**
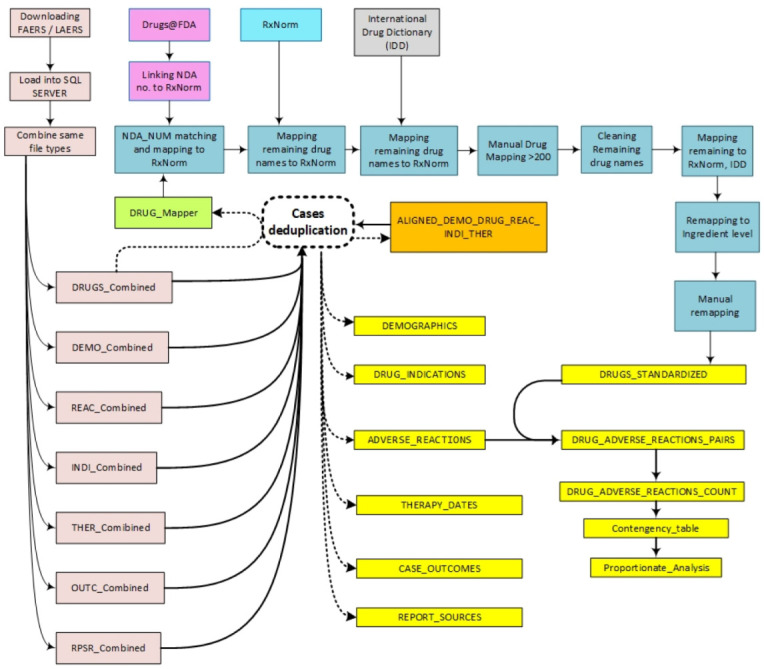
Steps of FAERS/LAERS dataset curation.

A graphical representation of all the steps involved in creating this dataset is illustrated in Figure 2.

### 2.4. RxNorm 

A comprehensive drug vocabulary to normalize and standardize all the drug names in the dataset to the single active ingredient level is needed, so that all the same drug entities but with different formats should be collapsed into only one from.

RxNorm is a standard clinical drug vocabulary that includes other source vocabularies, in addition to the RxNorm source vocabulary [21]. RxNorm also provides relationships between drug names, which would help normalize drug names to a set target, the active ingredient level.

Moreover, RxNorm is open to the public after proper user registration. All these factors make RxNorm the best choice for performing this task.

### 2.5. Drug Names Mapping

All “DRUG” files from the FAERS/LAERS database were merged into a “DRUG_combined” table. Then, the provided New Drug Application (NDA) number in the “DRUG_combined” table was used to map drug names to FDA-approved drugs database (Drugs@FDA), which was already mapped to RxNorm. Drugs@FDA can be downloaded from: https://www.fda.gov/drugs/drug-approvals-and-databases/about-drugsfda (Accessed on 1 December 2021).

The next step was performing string matching for the remaining drug names in the “DRUG_combined” table with RxNorm, combined with minimal string cleaning and the removal of leading and trailing spaces and unexplained symbols that might be present in some drug names.

After the previous two steps, only 8% of drug names remained unidentified, which included trade names that are not registered in the United States of America and drugs with different naming conventions (i.e., a different sequence or combination of the drug names, dosage forms, strengths, units, or manufacturer names) than the standard RxNorm vocabulary. They also include misspelled drug names, devices, non-specific drug names or drug categories, and in some cases, unidentified texts. Examples of these remaining drug names are shown in Table 2.

To identify as much as possible from the unidentified drug names, the following steps were performed in order:

1. The International Drug Dictionary (IDD) [22] was used to map drug trade names of multinational origin directly to RxNorm.
2. Manual drug mapping for drug names counted >200.
3. Extensive text cleansing of the remaining unidentified drug names, followed by a second time string matching with RxNorm, then with the IDD.

### 2.6. Drug Names Normalization

RxNorm also provides relationships between drug names, which would help normalize drug names to the set target, the single active ingredient level. Unlike Banda et al.’s dataset, where the multi-ingredient drugs are kept as one entity, the authors have separated the multi-drug entities into single active ingredients in the dataset. This approach creates more accurate counts, and subsequently, more accurate signal detection, of drug-adverse event pairs. An active ingredient in a multi-ingredient dosage form within the dataset will not be readily accessible for inclusion in the total count of the same active ingredient originating from the other single and multi-ingredient forms within the dataset. The background counts in the contingency table for the “all other drugs” will also be affected.

The multi-ingredient drugs are still traceable in the dataset, through the “Drug_id” serial numbers which were given to all drug entries in the “DRUG_combined” table. In cases in which a multi-ingredient form is separated into single ingredients, the newly separated entities have the same parent multi-ingredient “Drug_id” number.

The mapping logic in the previous step was primarily targeting RxNorm as the primary source vocabulary. If there is no match with RxNorm, then it will try to match other source vocabularies. However, even those mapped to RxNorm source vocabulary are not all at the active ingredient level. Therefore, all drugs that were not mapped to the source vocabulary RxNorm in the single active ingredient level need to be remapped to the RxNorm source vocabulary as single drug entities at the ingredient level. The RxNorm relationships was used to do the remapping to the single active ingredient level.

Finally, the authors performed a manual remapping step to maximize the number of normalized drugs to the ingredient level in RxNorm.

A few exceptions were made for some drugs, which included:

1. When other source vocabularies express the active components more precisely (in terms of granularity) than the RxNorm, then the other vocabulary name is adapted.
2. All vaccines are kept in their best string matching the RxNorm, regardless of whether they match RxNorm source vocabulary or other source vocabularies, and also might contain combined vaccines.
3. Active ingredients that are not included in the RxNorm source vocabulary but are maintained in other source vocabularies are mapped to those other vocabularies.

However, all drug names in the dataset are mapped to a unique identification number for each entity called the Atom Unique Identifier (RXAUI) from RxNorm, representing about 97.1% of all drug names in the FAERS/LAERS database.

### 2.7. Creating Drug-Adverse Reaction Contingency Table

A drug-adverse reaction contingency table was created, based on the concept of Table 3, that would serve as the backbone for any statistical or disproportional analysis. A total of 6,325,800 drug-adverse event aggregates composed the contingency table for the dataset.

### 2.8. Data Mining

To further make it easier for researchers who are not familiar with large databases, a “ready to use” proportional analysis table was made for each drug-adverse event pair in the dataset. The reporting odds ratio (ROR) [23], proportional reporting ratio (PRR) [24,25], and information component (IC) [26,27] were considered as the data-mining tools to be incorporated in the proportionate analysis table. These measures will return a higher score when a particular drug is associated with a specific adverse event more than expected (i.e., more than other drugs). For the uncertainty estimation, the Chi-squared analysis with Yates correction ($x^{2}$) [28,29] and the 95% confidence intervals (CI) of ROR, PRR, and the IC were also included.

$$\mathrm{PRR}=\frac{a/\left(a+c\right)}{b/\left(b+d\right)}$$

$$\mathrm{PRR}\;95\%\;\mathrm{CI}=e^{\ln\left(PRR\right)\pm 1.96\sqrt{\left(\frac{1}{a}-\frac{1}{a+c}+\frac{1}{b}-\frac{1}{b+d}\right)}}$$

$$\mathrm{ROR}=\frac{\left(a/c\right)}{\left(b/d\right)}=\frac{ad}{bc}$$

$$\mathrm{ROR}\;95\%\;\mathrm{CI}=e^{\ln\left(ROR\right)\pm 1.96\sqrt{\left(\frac{1}{a}+\frac{1}{b}+\frac{1}{c}+\frac{1}{d}\right)}}$$

$$x_{yates}^{2}=\frac{\left(a+b+c+d\right)\times {\left(\left|\left(a\times d\right)-\left(b\times c\right)\right|-\left(a+b+c+d\right)/2\right)}^{2}}{\left(a+c\right)\times \left(a+b\right)\times \left(b+d\right)\times \left(d+c\right)}$$

$$\mathrm{Information}\;\mathrm{component}\;\left(IC\right)=\log_{2}\frac{a+0.5}{a_{exp}+0.5}$$

$$a_{exp}=\frac{\left(a+b\right)*\left(a+c\right)}{\left(a+b+c+d\right)}$$

$$IC_{025}=IC-3.3\times {\left(a+0.5\right)}^{-1/2}-2\times {\left(a+0.5\right)}^{-3/2}$$

$$IC_{975}=IC+2.4\times {\left(a+0.5\right)}^{-1/2}-0.5\times {\left(a+0.5\right)}^{-3/2}$$

### 2.9. Dataset Validation 

First, the method of importing the data files was validated by checking the counts of records of 10 imported tables and found them to be consistent with the counts stated with the relevant raw files. Additionally, the first and the last ten records in these ten tables were verified manually and found to be consistent with the relevant records in the original raw data files.

Second, manual auditing was performed for 20 randomly selected case records from the final deduplicated dataset to confirm that:

1. Demographic files have been linked correctly to the drugs, indications, reactions, and therapy start date for each case report;
2. There is no case duplication for the data that have been selected.

Third, another 20 randomly selected records were sampled from the dropped case records for being duplicated and verified that:

1. The deduplication matching criteria for these case reports have been fulfilled;
2. These dropped cases are those that should be dropped based on the applied criteria.

Fourth, 50 records were randomly selected from the final standardized drug table and the following were manually verified:

1. Active ingredients have been retrieved correctly for each drug name;
2. The RXAUI mapping to the source drug names was done correctly;
3. Adverse reactions for each drug have been retrieved correctly in the drug-adverse reaction pairs table.

### 2.10. Code Availability

The codes used to create the dataset are publicly available (https://github.com/Mohammad-Ali-Khaleel/FAERS).

## 3. Results

### Dataset Created

The cleaned, deduplicated, and standardized dataset created from FAERS/LAERS data files consists of eleven tables; each has been exported into a text file, and all files are compressed into a single zip file. The SQL Server code for installing these tables into SQL Server and a data dictionary file describing the fields in the data files have been provided within the same zip file.

These files are as follows:

1. DEMOGRAPHICS: This file contains the basic patients’ demographic data extracted from all the DEMO files. The reporting country code or “COUNTRY_CODE” has been standardized in this table to the ISO 3166 standard codes. Moreover, the age field has been normalized to represent values in years.
2. DRUGS_STANDARDIZED: Extracted from the merged DRUG files (“DRUG_combined” file), with drug names being mapped to the single active ingredient level and standardized to RxNorm. A drug identification number “DRUG_ID” field was added to the raw data before processing the “DRUG_combined” file to ensure that combined drug active in the same dosage form will have the same “DRUG_ID” when they become separate entities in the final “DRUGS_STANDARDIZED” file, so that it will be easy to trace multi-ingredient drugs in the dataset if needed.
3. ADVERSE_ REACTIONS: This file includes the adverse events associated with each case report in the dataset and created from combining “REAC” files.
4. DRUG_ADVERSE_REACTIONS_PAIRS: This file is composed of all adverse drug reactions paired with the associated drug name.
5. DRUG_ADVERSE_REACTIONS_COUNT: This file includes drugs associated with adverse reactions and frequency counts for each association.
6. DRUG_INDICATIONS: This file includes the combined “INDI” files from FAERS/LAERS database. Entries with drug indication descriptions as “Product used for the unknown indication” and “Drug use for the unknown indication” have been considered as null values and thereby deleted.
7. CASE_OUTCOMES: This file contains the patient outcome details. It was created from the combined “OUTC” files.
8. THERAPY_DATES: Contains information about the start and end therapy dates and also the duration of the therapy. It was created from the “THER” files.
9. REPORT_SOURCES: Contains information about the source of the reports. It was created from the “RPSR” files.
10. CONTINGENCY_TABLE: This file contains the 2 X 2 table or contingency table for drugs and adverse drug reactions.
11. PROPORTIONATE_ANALYSIS: This file contains the pre-calculated proportionate statistics, the ROR, PPR, and IC with upper and lower 95% confidence intervals. It also contains the Chi-squared analysis with Yates correction ($x^{2}$).

**Figure 3:**
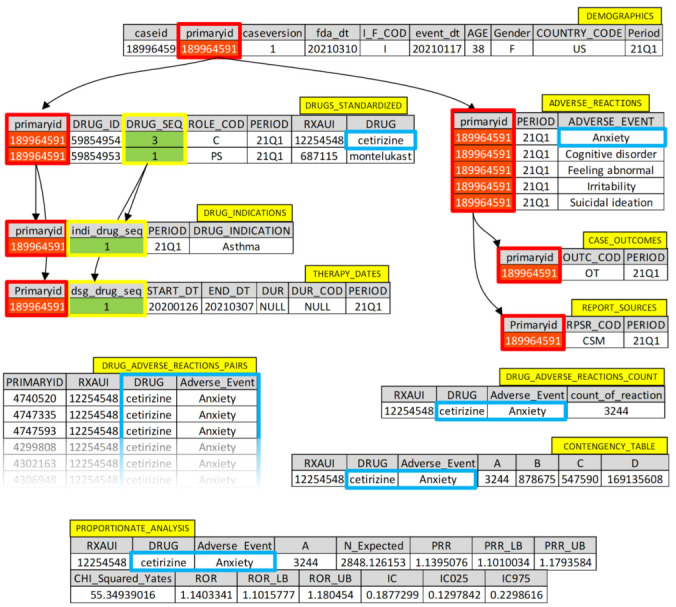
Detailed example of data retrieval from the dataset.

A detailed example of data retrieval from the dataset in illustrated in Figure 3.

## 4. Conclusions

The verification steps and the intensive work carried out to create this dataset ensures the quality and consistency of the produced dataset to be valid for spontaneous adverse drug reactions data analysis.

This dataset can be considered as a reliable and accurate source of pharmacovigilant data mining and hypothesis generation regarding drug post-marketing safety surveillance acquired from the FAERS/LAERS data files for the period from January 2004 to September 2021.

## Figures and Tables

**Table 1:** Top 20 reporting countries to FAERS/LAERS database in descending order.

Reporter Country	%
United States of America	64.91%
United Kingdom	3.61%
Japan	3.32%
Canada	3.26%
France	3.15%
Germany	2.44%
Italy	1.44%
Brazil	1.04%
Spain	0.86%
Australia	0.82%
The Netherlands	0.73%
China	0.70%
Switzerland	0.38%
Sweden	0.37%
India	0.36%
Colombia	0.35%
Turkey	0.29%
Belgium	0.28%
Argentina	0.28%
Poland	0.26%

**Table 2:** Examples of unidentified entries after exact string matching with RxNorm.

Drug Name
BLOOD THINNER
CLINICAL TRIAL PILL
FT?2102
CC-292
...
[COMPOSITION UNSPECIFIED]
NO SUBJECT DRUG
RIBAVARIN
ADDITIONAL STUDY MEDICATION
ALL OTHER THERAPEUTIC PRODUCTS (ALL OTHER THERAPEUTIC PRODUCTS)
BIRTH CONTROL PILL
Drug name unspesified
IBUROFEN
ANTIBIOTICS (ANTIBIOTICS)
Allergy medication
AMITRIPTLINE
LAMOTRGINE
PREPARATION H NOS
CPAP MACHINE
NO MATCH
TERCIAN TABLETS
LOSEC I.V.
LOXOPROFEN SODIUM (LOXOPROFEN SODIUM)
PHENERGAN TABLETS/SUPPOSITORIES
HYDROCHLOROTHIAZIDE AND RAMIPRIL
GALANTAMINE 4MG
LESCOL ^SANDOZ^
EPITOMAX (TOOPIRAMATE) TABLETS
ACETAMINOPHEN (LONG-ACTING)()
VALSARTAN-TABLET-UNIT DOSE: UNKNOWN
CLOPIDOGREL/ASPIRIN) -
CALCIUM & VITAMIN D /01483701/
LIDOCAINE HYDROCHLORIDE;PRILOCAINE HYDROCHLORIDE

**Table 3:** Contingency table.

	Drug X	All Other Drugs	Total
Adverse event Y	a	b	a + b
All other adverse events	c	d	c + d
Total	a + c	b + d	a + b + c + d

a = The number of reports of the drug of interest (X) with the adverse event of interest (Y). b = The number of reports of all other drugs with the adverse event of interest (Y). c = The number of reports of the drug of interest (X) with all other adverse events. d = The number of reports of all other drugs with all other adverse events.

## Data Availability

The dataset presented in this study is openly available in the Mendeley data repository at (https://doi.org/10.17632/73p7w6gvtt.1).
